# High-Definition Transcranial Direct Current Stimulation of the Dorsolateral Prefrontal Cortex Modulates the Electroencephalography Rhythmic Activity of Parietal Occipital Lobe in Patients With Chronic Disorders of Consciousness

**DOI:** 10.3389/fnhum.2022.889023

**Published:** 2022-05-27

**Authors:** Jinying Han, Chen Chen, Shuang Zheng, Xiaoxiang Yan, Changqing Wang, Kai Wang, Yajuan Hu

**Affiliations:** ^1^Department of Neurology, The First Affiliated Hospital of Anhui Medical University, Hefei, China; ^2^Collaborative Innovation Center of Neuropsychiatric Disorders and Mental Health, Hefei, China; ^3^Anhui Province Key Laboratory of Cognition and Neuropsychiatric Disorders, Hefei, China; ^4^The School of Mental Health and Psychological Sciences, Anhui Medical University, Hefei, China; ^5^Hefei Comprehensive National Science Center, Institute of Artificial Intelligence, Hefei, China

**Keywords:** disorders of consciousness (DOC), electroencephalography (EEG), high-definition transcranial direct current stimulation (HD-tDCS), power spectral density (PSD), coma recovery scale-revision (CRS-R)

## Abstract

**Background:**

Disorders of consciousness (DOC) are a spectrum of pathologies affecting one’s ability to interact with the external world. At present, High-Definition Transcranial Direct Current Stimulation (HD-tDCS) is used in many patients with DOC as a non-invasive treatment, but electrophysiological research on the effect of HD-tDCS on patients with DOC is limited.

**Objectives:**

To explore how HD-tDCS affects the cerebral cortex and examine the possible electrophysiological mechanisms underlying the effects of HD-tDCS on the cerebral cortex.

**Methods:**

A total of 19 DOC patients were assigned to HD-tDCS stimulation. Each of them underwent 10 anodal HD-tDCS sessions of the left dorsolateral prefrontal cortex (DLPFC) over 5 consecutive days. Coma Recovery Scale-Revision (CRS-R) scores were recorded to evaluate the consciousness level before and after HD-tDCS, while resting-state electroencephalography (EEG) recordings were obtained immediately before and after single and multiple HD-tDCS stimuli. Depending on whether the CRS-R score increased after stimulation, we classified the subjects into responsive (RE) and non-responsive (N-RE) groups and compared the differences in power spectral density (PSD) between the groups in different frequency bands and brain regions, and also examined the relationship between PSD values and CRS-R scores.

**Results:**

For the RE group, the PSD value of the parieto-occipital region increased significantly in the 6–8 Hz frequency band after multiple stimulations by HD-tDCS. After a single stimulation, an increase in PSD was observed at 10–13 and 13–30 Hz. In addition, for all subjects, a positive correlation was observed between the change in PSD value in the parieto-occipital region at 10–13 and 6–8 Hz frequency band and the change in CRS-R score after a single stimulation.

**Conclusion:**

Repeated anodal HD-tDCS of the left DLPFC can improve clinical outcomes in patients with DOC, and HD-tDCS-related increased levels of consciousness were associated with increased parieto-occipital PSD.

## Introduction

Patients with Disorders of Consciousness (DOC) pose challenges to neurologists in terms of diagnosis, prognosis, and medical treatment (Giacino, 2005). Based on neurobehavioral function, DOC include a wide spectrum of pathologic consciousness which can be subdivided into unresponsive wakefulness syndrome [UWS, formally called vegetative state (VS)], minimally conscious state (MCS) and emergence of the minimally conscious state (EMCS). VS patients show only reflexive movements and behavioral evidence for self- or environmental awareness is completely absent (Laureys et al., 2010). In contrast, MCS patients show fluctuating but reproducible signs of consciousness (Giacino et al., 2002). In EMCS, patients are able to communicate functionally and/or use objects (Giacino et al., 2002). Considering the increase in the number of individuals affected by this disease and the socioeconomic burden it brings, there is still lack of effective standardized treatment for DOC patients.

Electroencephalography (EEG) is a widely accepted method for obtaining specific information on the level of cortical information processing and changes (Syczewska and Zielińska, 2010; Bai et al., 2021). Changes in brain function caused by DOC can be measured by EEG, which may reflect the level of information processing and integration in the cortex (Bagnato et al., 2015; Bai et al., 2017b). Studies on VS patients have reported neuroimaging evidence of a drop in global cortical metabolism during the resting state (Schiff et al., 2002). It has been previously reported that the degree of DOC may be correlated with increased low-frequency band power in the EEG compared with the normal population (Leon-Carrion et al., 2008). Recent research on EMCS patients has found that certain brain areas show a higher degree of metabolism recovery than others. These brain regions coincide with the multimodal associative cortex, namely the prefrontal, parieto-temporal, and posterior parietal regions (Laureys et al., 1999; Laureys, 2005), which play an important part in higher cortical functions.

In addition to deep brain stimulation and spinal cord stimulation, Transcranial Direct Current Stimulation (tDCS) has also been increasingly used to treat patients with DOC (Thibaut et al., 2015; Estraneo et al., 2017; Bai et al., 2018). High-Definition transcranial direct current stimulation (HD-Tdcs) is a non-invasive stimulation technique that uses small and specially arranged electrodes (Villamar et al., 2013). HD-tDCS with greater focus on target cortex and higher target intensity compared to traditional tDCS (Dmochowski et al., 2011). Because of its less-invasive impact, portability and economy, tDCS has become a valuable brain-intervention technique for rehabilitating DOC patients. The tDCS involves the application of a weak direct current into the cerebral cortex. Anodal tDCS has excitatory effects, while cathodal tDCS has inhibitory effects on the underlying cortex. Some studies have reported localized skin damage, mild burning, or mild pain after tDCS (Kessler et al., 2012). Numerous studies have demonstrated its efficacy in modulating the brains of patients with DOC (Hermann et al., 2020; Martens et al., 2020; Shou et al., 2021). It has been shown that tDCS can effectively modulate cortical excitability in patients with DOC (Bai et al., 2017a). These encouraging results were found by targeting the left dorsolateral prefrontal cortex (DLPFC) (Zhang and Song, 2018), but the underlying mechanisms of its effects on the cerebral cortex remain unclear.

In this study, we measured the resting state EEG before and immediately after HD-tDCS modulation. The power spectral density (PSD) was used to evaluate the effects of DOC on the brains of DOC patients and different frequency parameters of HD-tDCS were tested. In this way, we attempted to explore the possible mechanisms underlying the effects of HD-tDCS on the brain and to find evidence to support the utility of HD-tDCS in modulating the brains of DOC patients.

## Experimental Procedures

### Patients

A total of 19 DOC patients, aged 18–74 years old, who received anodal tDCS were enrolled in a consecutive session. Table 1 shows the clinical features of the 19 participants who completed the entire study. The consciousness state of each patient was assessed by two trained physicians using the CRS-R (Cai et al., 2019). The inclusion criteria for this study were (1) the patient was aged between 18 and 75 years and diagnosed with VS/MCS according to the CRS-R score; (2) the patient had stable vital signs even in the intensive care unit; (3) no neuromuscular blockers and sedatives were used within 24 h before participating in the study; (4) no improvement in the state of consciousness was observed within 1 week before the start of the study; and (5) the duration of the consciousness disturbance was at least 28 days. Exclusion criteria were (1) known preexisting severe neurocognitive degenerative disease; (2) head metal implantation; (3) previous craniotomy; (4) previous history of epilepsy; and (5) Previous treatment with transcranial electrical stimulation or transcranial magnetic stimulation.

**TABLE 1:** Demographic details for the patients.

ID	Sex	Age	Etiology	Days	T0 (CRS-R) (A-Vi-M-Ve-C-Ar)	T0-Clinical	T1 (CRS-R) (A-Vi-M-Ve-C-Ar)	T1-Clinical	T2 (CRS-R) (A-Vi-M-Ve-C-Ar)	T2-Clinical

						**Diagnosis**		**Diagnosis**		**Diagnosis**
RE 1	M	52	Trauma	84	11 (1/3/4/1/1/1)	MCS +	11 (1/3/4/1/1/1)	MCS +	12 (2/3/4/1/1/1)	MCS +
RE 2	F	49	HIE	30	6 (1/1/3/0/0/1)	MCS −	7 (2/1/3/0/0/1)	MCS −	15 (3/3/5/2/1/1)	MCS +
RE 3	M	53	Trauma	34	5 (0/0/5/0/0/0)	MCS −	7 (0/0/5/1/0/1)	MCS −	14 (3/2/5/2/1/1)	MCS +
RE 4	F	74	Hemorrhage	101	11 (2/3/3/0/1/2)	MCS +	11 (2/3/3/0/1/2)	MCS +	15 (3/4/5/0/1/2)	MCS +
RE 5	M	49	Hemorrhage	50	5 (1/1/1/0/0/2)	VS	6 (1/1/1/1/0/2)	VS	7 (1/1/2/1/0/2)	VS
RE 6	M	55	Trauma	302	6 (1/2/1/0/0/2)	VS	6 (1/2/1/0/0/2)	VS	8 (2/3/1/0/0/2)	MCS −
RE 7	M	72	Cerebral infarction	42	5 (1/3/0/0/0/1)	MCS −	5 (1/3/0/0/0/1)	MCS −	9 (2/3/0/2/1/1)	MCS +
RE 8	M	47	Hemorrhage	29	6 (1/1/2/1/0/1)	VS	6 (1/1/2/1/0/1)	VS	12 (2/3/3/1/1/2)	MCS +
RE 9	M	58	Trauma	53	9 (2/3/2/0/0/2)	MCS −	9 (2/3/2/0/0/2)	MCS −	10 (2/3/2/1/0/2)	MCS −
RE 10	F	68	Hemorrhage	30	8 (2/1/3/0/0/2)	MCS −	9 (3/1/3/0/0/2)	MCS +	15 (3/4/5/0/0/3)	MCS +
RE 11	M	59	Cerebral infarction	68	5 (2/1/0/0/0/2)	VS	5 (2/1/0/0/0/2)	VS	6 (3/1/0/0/0/2)	MCS +
N-RE 1	M	54	Hemorrhage	73	6 (1/1/2/0/0/2)	VS	6 (1/1/2/0/0/2)	VS	6 (1/1/2/0/0/2)	VS
N-RE 2	M	56	HIE	41	2 (0/0/0/0/0/2)	VS	2 (0/0/0/0/0/2)	VS	2 (0/0/0/0/0/2)	VS
N-RE 3	F	39	HIE	128	4 (0/0/2/0/0/2)	VS	4 (0/0/2/0/0/2)	VS	4 (0/0/2/0/0/2)	VS
N-RE 4	M	18	Disseminated cerebrospinal meningitis	48	4 (1/1/0/0/0/2)	VS	4 (1/1/0/0/0/2)	VS	4 (1/1/0/0/0/2)	VS
N-RE 5	M	56	Hemorrhage	88	3 (0/0/1/0/0/2)	VS	3 (0/0/1/0/0/2)	VS	3 (0/0/1/0/0/2)	VS
N-RE 6	M	64	Hemorrhage	34	10 (1/1/5/0/1/2)	MCS −	10 (1/1/5/0/1/2)	MCS −	10 (1/1/5/0/1/2)	MCS −
N-RE 7	F	70	Cerebral infarction	58	4 (1/1/0/1/0/1)	VS	4 (1/1/0/1/0/1)	VS	4 (1/1/0/1/0/1)	VS
N-RE 8	F	39	HIE	215	3 (0/0/0/1/0/2)	VS	3 (0/0/0/1/0/2)	VS	3 (0/0/0/1/0/2)	VS

*RE, responsive group; N-RE, non-responsive group; HIE, hypoxic-ischemic encephalopathy; VS, vegetative state; MCS, minimally conscious state; CRS-R, Coma Recovery Scale-Revised; CRS-R subscales: A, auditory function; Vi, visual function; M, motor function; Ve, verbal; C, communication; A, arousal; T0, before the experiment; T1, after a single session of HD-tDCS; T2, after treatment for 5 days.*

Written informed consent to participate in the study was obtained from the patient’s caregivers, and the study was approved by the ethics committee of the First Affiliated Hospital of Anhui Medical University.

### Stimulation Protocol

**FIGURE 1:**
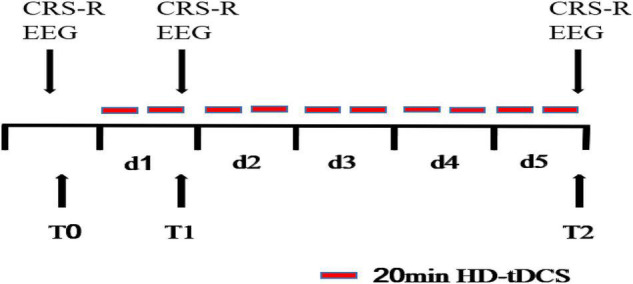
tDCS procedure.

**FIGURE 2:**
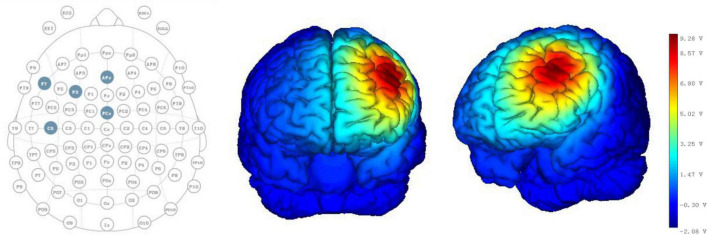
HD-tDCS electrode positions. F3 represents the anodal electrode, and AFz, FCz, F7, and C5 represent cathodal electrodes. Stimulated distribution of the electric field in cortical gray matter with the selected montage.

All patients received HD-tDCS modulation for two session per day over 5 consecutive days (Figure 1). As shown in Figure 2, the anodal HD-tDCS electrode was placed over the left DLPFC (F3 in the 10–20 international system EEG placement), and four cathodal electrodes were placed centered over AFz, FCz, F7, and C5 approximately 3–5 cm away from the center electrode (Neuroelectrics, Barcelona, Spain). The stimulation was a constant current of 2 mA applied for 20 min with a 30 s fade in/fade out period. Based on the finite element model (FEM) head model (Bikson and Datta, 2012; Datta et al., 2013), the stimulation parameters in this study (such as stimulation site, size, and input current) were used to calculate the theoretical current/electric intensity and distribution on the cortex. Figure 2 shows the visualization of stimulated distribution of the HD-tDCS electric field in cortical gray matter with the selected montage. The CRS-R assessments and EEG recordings were performed at three time points: before the experiment (T0), after a single session of HD-tDCS (T1), and after treatment for 5 days (T2).

### Electroencephalography Recordings and Pre-processing

EEG data were continuously recorded from 19 channels at positions of the International 10/20 system for at least 6 min (EEG-1200C, Nihon Kohden, Shinjuku, Japan). The EEG data were digitized at a sampling rate of 200 Hz. The electrode impedance was maintained below 5 kΩ. EEG data were recorded using the average of C3 and C4 as the system reference, and Nz as the ground electrode.

EEG pre-processing was performed using EEGLAB 13.0b, running on a MATLAB environment (Version 2013b, MathWorks Inc.; Natick, MA, United States). The EEG data were filtered using a bandpass of 0.1–40 Hz and notch filter of 48–52 Hz. Then the extract of continuous EEG data from 20 to 320 s was segmented into 2-s-long epochs. The independent component analysis function was used to identify and remove the artifact-relevant components, including eye movements and muscle activation. EEG data were average-referenced and rejected epoch when more than ± 150 μv.

### Electroencephalography Analysis

In this study, the subjects were divided into RE and N-RE groups according to whether the CRS-R score changed after HD-tDCS stimulation. In addition, the study also divided the brain into four regions, namely, left frontal lobe, right frontal lobe, central, and parieto-occipital regions (Wu et al., 2019). The left frontal region included electrodes Fp1, F3, and F7; the right frontal region included electrodes Fp2, F4, and F8; the central region included electrodes Cz, C3, and C4; and the parieto-occipital region included electrodes Pz, P3, P4, O1, and O2. Absolute power spectrum density value (μV^2^/Hz) (Thatcher et al., 2005; El-Hagrassy et al., 2021) in each frequency band (delta: 0.1–4 Hz; theta1: 6–8 Hz; theta2: 8–10 Hz; alpha1: 8–10 Hz; alpha2: 10–13 Hz; beta: 13–30 Hz) (Tarasova et al., 2019) were computed using fast Fourier transform with the Welch method (Welch, 1967).

### Statistical Analysis

Statistical analysis used SPSS 19.0 (IBM, Armonk, NY, United States) to compare the demographic information and resting-state EEG data between RE and N-RE groups. Numerical variables were expressed as means ± standard deviation and tested for normal distribution before comparison. Baseline characteristics between the two groups were compared using the χ^2^-test for categorical variables, and the t- or Mann-Whitney *U*-tests for continuous variables paired *t*-test was used to analyze the PSD at different time. Spearman’s rank test was calculated to test the correlation between the change in PSD values and increased CRS-R scores of the DOC patients. *p* < 0.05 was considered statistically significant.

## Results

### Demographic and Behavioral Results

**FIGURE 3:**
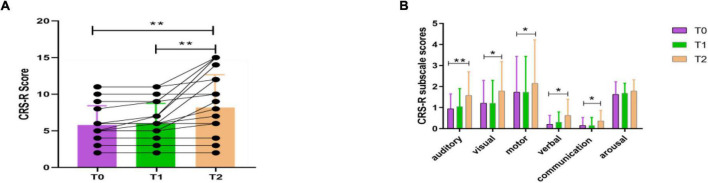
CRS-R total scores **(A)** and CRS-R subscale scores **(B)** for all patients before the experiment (T0), after a single session of HD-tDCS (T1) and after treatment for 5 days (T2). CRS-R, Coma Recovery Scale-Revised; *p < 0.05, **p < 0.01.

In this study, we included a total of 19 patients (8 MCS, 11 VS). Among them, there were eight males and three females in the RE group, and five males and three females in the N-RE group. The mean age was 57.81 ± 9.51 years for the RE group and 49.5 ± 16.68 years for the N-RE group. As for the onset time, the average duration was 74.81 ± 78.98 days for the RE group and 85.62 ± 60.39 days for patients in N-RE group. In the RE group, the etiology included trauma (4), hypoxic ischemic encephalopathy (1), cerebral hemorrhage (4), and cerebral infarction (2). In the N-RE group, etiologies included fulminant encephalomyelitis (1), hypoxic ischemic encephalopathy (3), cerebral hemorrhage (3), and cerebral infarction (1). The RE group and N-RE group did not differ in time from onset (*z* = −1.033, *p* = 0.302), age (*t* = −1.382, *p* = 0.185), sex (*z* = −0.461, *p* = 0.717), and etiologies (χ^2^ = 5.807, *p* = 0.191). After one session of stimulation, we observed that CRS-R scores increased in four patients (21.05%; 3 MCS, 1 VS). After 10 sessions of stimulation, we observed that CRS-R scores increased in 11 patients (57.89%; 7 MCS, 4 VS). No decrease in CRS-R score was observed in any patient after stimulation. The CRS-R score at T2 was significantly higher than at T0 (*t* = −3.350, *p* = 0.004; Figure 3A), whereas CRS-R score changes were not significantly different between T0 and T1 (*t* = −2.041, *p* = 0.056). We also examined the CRS-R subscale scores, which revealed a significant improvement in the auditory (*t* = −3.314, *p* = 0.004), visual (*t* = −2.625, *p* = 0.017), motor (*t* = −2.388, *p* = 0.028), verbal (*t* = −2.388, *p* = 0.028), and communication scores (*t* = −2.191, *p* = 0.042) after repeated stimulation (Figure 3B).

### Power Spectrum Density Results

**FIGURE 4:**
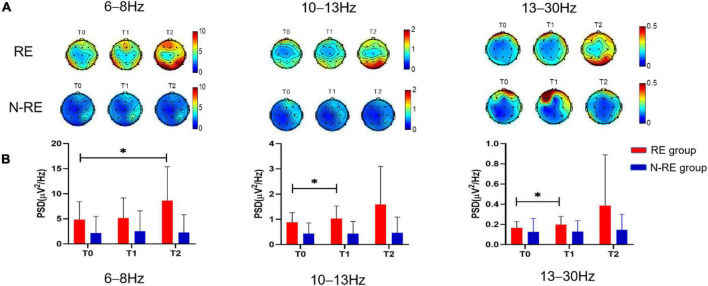
The PSD topographic map of the RE group and the N-RE group **(A)**, and the PSD of the RE group and the N-RE group of the parieto-occipital region **(B)** before the experiment (TO), after a single session of Tdcs (TI) and after the treatment of 5 days (T2). PSD power spectrum density, RE responsive group, N-RE non-responsive group. *p < 0.05.

For the RE group, in the parieto-occipital region, the PSD at T2 was significantly higher than at T0 (from 4.82 to 8.64, *t* = −2.261, *p* = 0.047, effect size = 0.6817; 95%CI, 0.0078-1.3286, Figure 4), whereas PSD was not significantly different between T0 and T1 at 6–8 Hz (from 4.82 to 5.17, *t* = −0.678, *p* = 0.513, effect size = 0.2044; 95%CI, −0.3981 to 0.7970). Compared with T0, the PSD at T1 showed a significant increase at 10–13 Hz (from 0.88 to 1.03, *t* = −2.572, *p* = 0.028, effect size = 0.775; 95%CI, 0.0816-1.4406, Figure 4) and at 13–30 Hz (from 0.16 to 0.19, *t* = −2.460, *p* = 0.034, effect size = 0.7417; 95%CI, 0.0552-1.4000, Figure 4), but this change at 10–13 Hz (from 0.88 to 1.59, *t* = −1.476, *p* = 0.171, effect size = 0.4450; 95%CI, −0.1862 to 1.0566) and at 13–30 Hz (from 0.16 to 0.38, *t* = −1.426, *p* = 0.184, effect size = 0.4300; 95%CI, −0.1994 to 1.0398) was not observed between T2 and T0.

For the N-RE group in the parieto-occipital region, the PSD showed no obvious differences when T2 and T1 were compared with T0 at 6–8 Hz (from 2.16 to 2.28, *t* = −1.169, *p* = 0.281, effect size = 0.1770; 95%CI, −0.3012 to 1.0585; from 2.16 to 2.52, *t* = −1.263, *p* = 0.247, effect size = 0.1993; 95%CI, −0.2748 to 1.0933, respectively), at 10–13 Hz (from 0.43 to with 0.46, *t* = −0.977, *p* = 0.357, effect size = 0.3257; 95%CI, −0.3556 to 0.9881; from 0.43 to 0.44, *t* = −0.152, *p* = 0.883, effect size = 0.0507; 95%CI, −0.6048 to 0.7029, respectively) or at 13–30 Hz (from 0.12 to 0.14, *t* = −1.098, *p* = 0.304, effect size = 0.3660; 95%CI, −0.3210 to 1.0322; from 0.12 to 0.13, *t* = −0.076, *p* = 0.942, effect size = 0.0253; 95%CI, −0.6290 to 0.6780, respectively) respectively.

**FIGURE 5:**
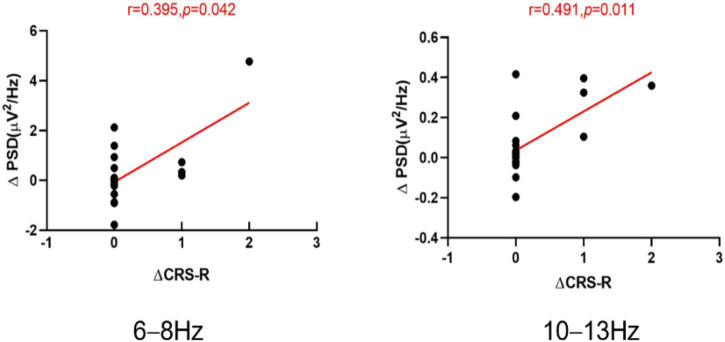
The scatter plots of the ΔCRS-R and ΔPSD at the 6–8 and 10–13 Hz band for all the patients, ΔCRS-R the increases in the Coma Recovery Scale-Revised (CRS-R) scores at T1 compared with T0. ΔPSD the increases in the power spectrum density (PSD) at T1 compared with T0. T0 before the experiment. T1 after a single session of tDCS.

In the parieto-occipital region, when compared with T0, the increases in the CRS-R at T1 showed a significant positive correlation with the change in PSD at 6–8 Hz (*r* = 0.395, *p* = 0.042; Figure 5) and the increases in the CRS-R at T1 showed a positive correlation (*r* = 0.491, *p* = 0.011; Figure 5) with the change in PSD at 10–13 Hz for all patients.

## Discussion

In this study, in order to explore the mechanism of HD-tDCS in patients with DOC, we investigated the changes in PSD in different brain regions for patients with DOC who received 5 consecutive days of HD-tDCS. Furthermore, we correlated the CRS-R scores with the power of each frequency band. We found that HD-tDCS in patients with DOC over the DLPFC improved not only the behavioral but also the neurophysiological signature of the EEG. After repeated HD-tDCS, the CRS-S score increased significantly. Moreover, the PSD of theta, alpha and beta bands in the parieto-occipital region was significantly increased in patients who responded to HD-tDCS. For non-responsive patients, no significant changes were seen in the PSD in any frequency band. This research thus demonstrated that the PSD of the theta and alpha bands was positively correlated with CRS-R scores in responding patients.

Changes in the EEG signature of conscious patients usually precede clinical behavioral manifestations. EEG signatures are frequently used to diagnose and assess prognosis in patients with DOC (Bagnato et al., 2010, 2015). According to one study, normalized delta power decreased from VS to a conscious state, while normalized theta and normalized alpha power in parietal regions increased significantly (Sitt et al., 2014). Due to varying degrees of functional deafferentation at thalamic and cortical levels, slow-wave activity predominates after severe brain injury (Schiff et al., 2014). In patients with large hemispheric infarctions, higher levels of consciousness were associated with more alpha and beta oscillations and fewer delta and theta oscillations (Huang et al., 2020). In the present study, a significant increase in alpha and beta power was observed in the parieto-occipital region of patients with impaired consciousness after a single stimulation, when clinical behavior was not yet significantly different. The above phenomena occurred only in those who responded to HD-tDCS. Similarly, another research study has shown that after active intervention of tDCS over the precuneus, MCS patients displayed improvements in local and global information processing of the beta and gamma bands in resting-state functional brain networks, which were distributed across nearly all brain regions (Zhang et al., 2020). Many studies have shown that changes in theta activity are related to working memory and consciousness (Gevins and Smith, 2000; Jensen and Tesche, 2002; Lehembre et al., 2012). Low and high alpha oscillations are the first to restore self-regulation after recovery of full consciousness and represent a turning point in the transition between unconsciousness and consciousness (Bonfiglio et al., 2014). High-alpha band (10–13 Hz) activity over posterior brain areas possibly reflects long-term memory activation (Sauseng et al., 2006). Three experiments comparing finger acceleration on a reaction time task of the stretch reflex showed that beta-band cortical synchronization increased the maintenance of the current motor state, but attenuated the speed of the new motor state, which was associated with Parkinson’s disease (Gilbertson et al., 2005). Beta oscillations are also involved in the long-range integration of cognitive functions (Engel and Fries, 2010; Donner and Siegel, 2011).

Consistent with previous studies, we found that theta power (Thibaut et al., 2018) and alpha power (Chennu et al., 2014; Cai et al., 2019) were significantly and positively correlated with behavioral scores based on the CRS-R. Several studies have confirmed that increased theta, alpha and beta power indicates a better state of consciousness (Sitt et al., 2014; Chennu et al., 2017; Huang et al., 2020). The effect of tDCS can also appear in other brain regions besides the stimulation site (Krause et al., 2017; Chan and Han, 2020). In our study, the stimulation site was in the DLPFC, but the final EEG PSD was significantly different in the parieto-occipital region. In the left prefrontal region, an upward trend in the PSD of each frequency band was also observed, but the change was not statistically significant. A study using fMRI confirmed that tDCS in the DLPFC increases the connectivity of the default mode network (internal awareness) and the left and right frontal-parietal networks (external awareness) at the stimulation site and in connecting brain regions (Keeser et al., 2011; Vanhaudenhuyse et al., 2011). Therefore, we speculate that the weak current signal through the skull increases the energy of the parieto-occipital region by increasing the connectivity of the fronto-parietal network, thereby regulating the state of consciousness of patients with impaired consciousness.

Some patients with DOC (21.05%) also responded to a single tDCS stimulation, which is consistent with Herman’s finding that 20% of patients showed behavioral improvements immediately after a single stimulation (Hermann et al., 2020). However, a study with 13 patients suggested that a single stimulation only increased the excitability of neurons in DOC patients with extensive damage to the cortical neural network, which was not sufficient to cause improvement in clinical behavior (Estraneo et al., 2017). Whether or not the CRS-R increased after a single stimulation compared to before stimulation, the onset time of the RE group was significantly shorter than that of the N-RE group. The responsiveness to tDCS is related to the time of onset (Estraneo et al., 2017), and shorter time to onset is indicative of better outcomes (Estraneo et al., 2020). Previous research has suggested that responsiveness to tDCS in patients with DOC is associated with residual brain metabolism, the structural integrity of cortical networks, and high connectivity of external control networks (Thibaut et al., 2015; Cavaliere et al., 2016; Bai et al., 2017a).

After a series of stimulations, the auditory, visual, motor, verbal, and communication subscale scores of CRS-R were increased, and similar results were also reported in another study (Zhang et al., 2017). At the same time, the number of responders (Thibaut et al., 2017) increased as the number of stimuli increased. During the follow-up, it was observed that a small number of patients gained the ability to act autonomously (such as eating bananas to know how to peel them, adjusting the position of an iPad to watch videos better). One patient in the RE group was diagnosed with EMCS half a month after stimulation, although his condition worsened about a month after stimulation, but was still better than before stimulation. Even more impressive was another study that followed a patient who was in VS for 6 years and then underwent a change in consciousness to MCS at follow-up 12 months after stimulation (Angelakis et al., 2014). There is an evidence showing that tDCS-induced distinct neural network participation patterns are temporally dispersed (Sehm et al., 2012). Responses to tDCS in patients with DOC can occur immediately after stimulation, but may also occur after a time delay. In general, non-synaptic effects lead to after-effects of tDCS in the human brain, possibly involving changes in transmembrane proteins and pH (Ardolino et al., 2005). Furthermore, numerous previous studies have found that the effect of tDCS lasts for at least 30 min (Boroda et al., 2020) and can extend out to 1 week (Thibaut et al., 2017) after the end of the stimulations. In fact, the effect of tDCS on neuroplasticity is time-dependent and degrades gradually over time (Boroda et al., 2020).

There are several limitations in this study. For one, the sample size was relatively small. We also observed that the PSD at 10–13 and 13–30 Hz in the parieto-occipital region, as well as the PSD in the left prefrontal region, showed an upward trend after multiple stimulations which was not statistically significant. In addition, the number of subjects classified according to etiology was relatively small, and the prognosis of different etiologies could not be effectively analyzed and compared. Finally, the 19-channel EEG signal captures only limited scalp information. At present, the mechanism of HD-tDCS in the treatment of patients with impaired consciousness is still unclear. The use of high-density EEG may help to determine the activation sequence of signals during the recovery process of patients and to analyze the activation of brain regions more accurately. Although there were numerous studies demonstrating the consciousness-improving effect of tDCS in patients with DOC, the spontaneous fluctuation of CRS-R are common cases for the MCS patients. In future studies, a sham control will be added and compared with the real HD-tDCS treatment, the number of patients will be expanded, the neurophysiological signals and prognosis differences between different causes of DOC will be analyzed, and the mechanism of action of HD-tDCS will be further explored.

## Conclusion

In this study, HD-tDCS was used to investigate the changes in PSD in DOC patients before and after intervention. In the RE group, the PSD values of the parieto-occipital region increased in multiple frequency bands regardless of single or multiple stimulations, but this change did not occur in the N-RE group. In addition, a positive correlation was found between the changes in CRS-R scores and the changes in PSD values in different frequency bands of the parieto-occipital region before and after HD-tDCS treatment in all patients. We speculate from this that HD-tDCS may modulate cerebral cortical excitability by changing the PSD of the parieto-occipital brain region, which provides a possible neural mechanism underlying the use of HD-tDCS for DOC patients from the perspective of neuroelectrophysiology.

## Data Availability Statement

The original contributions presented in the study are included in the article/supplementary material, further inquiries can be directed to the corresponding author/s.

## Ethics Statement

The studies involving human participants were reviewed and approved by the Ethics Committee of the First Affiliated Hospital of Anhui Medical University. The patients/participants provided their written informed consent to participate in this study.

## Author Contributions

JH and CC: full access to all data in the study and were responsible for integrity of the data and accuracy of the data analysis, acquisition, analysis, and interpretation of data. JH and YH: study concept and design. XY: collect, organize data, and follow up the subjects. YH: drafting of the manuscript and study supervision. JH: statistical analysis. YH and CW: application for funding. YH and SZ: administrative, technical, and material support. All authors: critical revision of the manuscript for important intellectual content.

## Conflict of Interest

The authors declare that the research was conducted in the absence of any commercial or financial relationships that could be construed as a potential conflict of interest.

## Publisher’s Note

All claims expressed in this article are solely those of the authors and do not necessarily represent those of their affiliated organizations, or those of the publisher, the editors and the reviewers. Any product that may be evaluated in this article, or claim that may be made by its manufacturer, is not guaranteed or endorsed by the publisher.
